# Supplementary material for: Content-rich biological network constructed by mining PubMed abstracts
Source: BMC Bioinformatics. 2004 Oct 8;5:147. doi: 10.1186/1471-2105-5-147 (PMC528731; doi:10.1186/1471-2105-5-147)
Supplement: Additional File 2 — The original results of the above study (non-essential files are deleted to keep the file size under the limit set by BMC bioinformatics). [file 1471-2105-5-147-S2.bz2 › chilibotAdditionalFile2/dip20/755ID8631671E18141/html/EXBB_EXBD.html]

 


 **EXBB** and **EXBD** 
  
Found 56 abstracts in PubMed, retrieved 20.  
 

 What does Google say? 
 PDF only 
| .edu only 

---

**Interactive relationship** (e.g. stimulation, inhibition, etc)

**Neutral relationship**- The aim of this review is to describe an in vivo assay of the interactions taking place in the Tol Pal or TonB  **ExbB**   **ExbD**  envelope complexes in the periplasm of Escherichia coli and between them and colicins or g3p protein of filamentous bacteriophages.  Ref: 12423784 Biochimie< MedlineTA>Biochimie,
- The recent observation that  **ExbB**  exists in 3.5 fold molar excess relative to the molarity of  **ExbD**  in E. coli suggests the possibility of two types of complexes, those containing both  **ExbB**  and  **ExbD**  and those containing only  **ExbB** .  Ref: 12193634 J Bacteriol, 2002
- Together, these results support an alternative model where all of the  **ExbB**  in the cell occurs in complex with all of the  **ExbD**  in the cell.  Ref: 12193634 J Bacteriol, 2002

**Non-interactive relationship** (e.g. studied together, co-existance, homology, etc.)

- **ExbB**  and  **ExbD** , despite being encoded from the same operon, were not equimolar, being present at 2463 522 and 741 105 copies respectively.  Ref: 11967085 Mol Microbiol, 2002
- and two cytoplasmic membrane proteins with less defined roles,  **ExbB**  and  **ExbD** .  Ref: 11967085 Mol Microbiol, 2002
- Following incubation on media containing hemoglobin, N. meningitidis IR3436 tonB  **exbB**   **exbD**  deletion mutant and N. gonorrhoeae PD3401 tonB insertional mutant give rise to colonies which can grow with hemoglobin.  Ref: 10986265 J Bacteriol, 2000
- The reconstitution in Escherichia coli of the S. marcescens Has system demonstrated that haem uptake is dependent on the E. coli  **ExbB** ,  **ExbD**  and TonB proteins.  Ref: 11737642 Mol Microbiol, 2001
- A putative Fur binding sequence located upstream from tonB was detected in a Fur titration assay, indicating that the tonB  **exbB**   **exbD**  operon may be Fur repressed in high iron growth conditions.  Ref: 10722583 Infect Immun, 2000
- A complex of three membrane spanning proteins TonB,  **ExbB**  and  **ExbD**  couples the chemiosmotic potential of the cytoplasmic membrane with siderophore uptake across the outer membrane.  Ref: 12409204 Biochim Biophys Acta, 2002
- Based on sequence similarities, the Bordetella pertussis tonB  **exbB**   **exbD**  locus was identified on a cloned DNA fragment.  Ref: 10722583 Infect Immun, 2000
- The ratio of these proteins was calculated at one TonB two  **ExbD**  seven  **ExbB**  under all four growth conditions tested.  Ref: 11967085 Mol Microbiol, 2002
- In gram negative bacteria, high affinity iron uptake requires the TonB  **ExbB**   **ExbD**  envelope complex to release iron chelates from their specific outer membrane receptors into the periplasm.  Ref: 10722583 Infect Immun, 2000
- Analysis of sequence alignments of TolQ and TolR homologues from numerous Gram negative bacterial genomes, together with analyses of the different tolQ tolR mutants, revealed that the TM domains of TolQ and TolR present structural and functional homologies not only to  **ExbB**  and  **ExbD**  of the TonB system but also with MotA and MotB of the flagellar motor.  Ref: 11722743 Mol Microbiol, 2001
- Downstream of comA two partially overlapping open reading frames termed  **exbB**  and  **exbD**  were found coding for putative proteins similar to proteins required for macromolecule uptake in Escherichia coli and present in other Gram negative bacteria.  Ref: 11446513 Res Microbiol, 2001
- In gram negative organisms, high affinity transport of iron substrates requires energy transduction to specific outer membrane receptors by the TonB  **ExbB**   **ExbD**  complex.  Ref: 12562789 J Bacteriol, 2003
- Pasteurella multocida  **exbB** ,  **exbD**  and tonB genes are physically linked but independently transcribed.  Ref: 12044675 FEMS Microbiol Lett, 2002
- The  **exbB** ,  **exbD**  and tonB genes of the Pasteurella multocida animal pathogen have been cloned by complementation of an Escherichia coli tonB mutant.  Ref: 12044675 FEMS Microbiol Lett, 2002
- Crystal structures of two outer membrane transport proteins, FhuA and FepA, and biochemical data reveal strong long range conformational changes of the proteins upon binding of Fe 3 siderophore complexes and in response to energy transfer from the cytoplasmic membrane into the outer membrane via the TonB  **ExbB**   **ExbD**  protein complex.  Ref: 11934617 Curr Opin Microbiol, 2002
- PCR approaches allowed the amplification, cloning and sequencing of A. suis tonB,  **exbB** ,  **exbD** , tbpB and tbpA homologues.  Ref: 12742718 Vet Microbiol, 2003
- Reverse transcription RT PCR, using RNA from organisms grown under iron replete and iron restricted conditions, revealed that tonB,  **exbB** ,  **exbD** , tbpB and tbpA are transcribed as a single unit with expression being up regulated in response to iron restriction.  Ref: 12742718 Vet Microbiol, 2003
- TonB, the energy transducer, appears to go through a cycle during energy transduction, with the absence of both  **ExbB**  and  **ExbD**  creating blocks at two points i in the inability of TonB to respond to the cytoplasmic membrane proton motive force and ii in the conversion of TonB from a high affinity outer membrane association to a high affinity cytoplasmic membrane association.  Ref: 12193634 J Bacteriol, 2002
- However, inducible levels of TX 100 resistance in gonococci had an absolute requirement for the TonB  **ExbB**   **ExbD**  system, suggesting that such resistance in gonococci has an energy requirement above and beyond that required for constitutive pump activity.  Ref: 11796379 Antimicrob Agents Chemother, 2002
- The Ton system consisting of the proteins TonB,  **ExbB**  and  **ExbD**  is required for colicin and microcin import.  Ref: 12423780 Biochimie< MedlineTA>Biochimie, 2002
- In this study, we report the per cell numbers of TonB,  **ExbB** ,  **ExbD**  and FepA for cells grown under iron replete and iron limited conditions.  Ref: 11967085 Mol Microbiol, 2002
- In Gram negative bacteria, the TonB  **ExbB**   **ExbD**  inner membrane multiprotein complex is required for active transport of diverse molecules through the outer membrane.  Ref: 11737642 Mol Microbiol, 2001
- lacZ promoter fusions confirmed that expression of the tonB2  **exbB**   **exbD**  genes is increased under conditions of iron limitation.  Ref: 10689178 FEMS Microbiol Lett, 2000
- They share homology with the  **ExbB** ,  **ExbD** , and TonB proteins, respectively.  Ref: 11418549 J Bacteriol, 2001
- Specifically, both  **ExbB**  and  **ExbD**  were found to be required for TonB to conformationally respond to proton motive force.  Ref: 12193634 J Bacteriol, 2002
- A second tonB gene in Pseudomonas aeruginosa is linked to the  **exbB**  and  **exbD**  genes.  Ref: 10689178 FEMS Microbiol Lett, 2000
- The energy is transduced to FecA by the inner membrane complex, TonB,  **ExbB** , and  **ExbD** .  Ref: 12618451 J Bacteriol, 2003
- **ExbB**  and  **ExbD**  proteins are part of the TonB dependent energy transduction system and are encoded by the exb operon in Escherichia coli.  Ref: 12193634 J Bacteriol, 2002
- , Triton X 100 TX 100 did not require the TonB,  **ExbB** , or  **ExbD**  protein.  Ref: 11796379 Antimicrob Agents Chemother, 2002
- The energy of the proton motive force is postulated to be transduced to the transport proteins by a protein complex that consists of the TonB,  **ExbB** , and  **ExbD**  proteins.  Ref: 11566987 J Bacteriol, 2001
- Both  **ExbB**  and  **ExbD**  were also required for association of TonB with the cytoplasmic membrane.  Ref: 12193634 J Bacteriol, 2002
- **ExbB**  and  **ExbD**  do not function independently in TonB dependent energy transduction.  Ref: 12193634 J Bacteriol, 2002
- Other biochemical evidence also shows that the proteins that provide energy for iron transport at the outer membrane, such as the TonB  **ExbB**   **ExbD**  system, are structurally very similar across bacterial species.  Ref: 11895294 Curr Top Med Chem, 2001
- Inducible, but not constitutive, resistance of gonococci to hydrophobic agents due to the MtrC MtrD MtrE efflux pump requires TonB  **ExbB**   **ExbD**  proteins.  Ref: 11796379 Antimicrob Agents Chemother, 2002
- Quantification of known components of the Escherichia coli TonB energy transduction system TonB,  **ExbB** ,  **ExbD**  and FepA.  Ref: 11967085 Mol Microbiol, 2002
- The last is involved in energy transduction between the inner and the outer membrane, and its conformation has been shown to depend on the presence of the proton motive force PMF,  **ExbB** , and  **ExbD** .  Ref: 11418549 J Bacteriol, 2001
